# Solriamfetol for the Use of Narcolepsy: A Systematic Review

**DOI:** 10.7759/cureus.24937

**Published:** 2022-05-12

**Authors:** Alisson Iturburu, Elisa Pallares Vela, Claudio Cruz, Mario Yepez, Juan Fernando Ortiz, Krithika Krishna, Gabriela Peña, Steven Cordova, Mahika Khurana, Pranathi Bandarupalli

**Affiliations:** 1 General Medicine, Universidad de Guayaquil, Guayaquil, ECU; 2 General Medicine, California Institute of Behavioral Neurosciences & Psychology, California, USA; 3 General Medicine, Universidad San Francisco de Quito, Quito, ECU; 4 Faculty of Health Sciences, Universidad Católica Santiago de Guayaquil, Guayaquil, ECU; 5 Neurology, Universidad San Francisco de Quito, Quito, ECU; 6 Neurology, Larkin Community Hospital, Miami, USA; 7 Pediatrics, Universidad San Francisco De Quito, Quito, ECU; 8 Public Health, University of California Berkeley, Berkeley, USA; 9 Neurology, Alluri Sitaramaraju Academy of Medical Sciences, Eluru, IND

**Keywords:** systematic review, pitolisant, excessive daytime sleepiness, cataplexy, narcolepsy, solriamfetol

## Abstract

Narcolepsy is a chronic and disabling neurological disorder characterized by excessive daytime sleepiness (EDS) and cataplexy. Historically, some medications have demonstrated efficacy in managing EDS and cataplexy symptoms. However, some patients cannot tolerate them, become refractory, or may use concomitant medications that preclude the use due to drug-drug interaction. Therefore, there is a necessity to explore the efficacy of new treatments, such as solriamfetol (JZP-110), a 2019 FDA-approved drug indicated to improve wakefulness in adults with EDS associated with narcolepsy.

We conducted this systematic review to investigate the effectiveness of solriamfetol in EDS and cataplexy, and the drug's overall safety. For this study, we used the PRISMA (Preferred Reporting Items for Systematic Reviews and Meta-Analyses) guidelines and MOOSE protocol. After an initial search of 119 papers, we included four clinical trials to investigate and analyze the use of solriamfetol for the treatment of narcolepsy.

Solriamfetol was proven to improve objective measures of EDS in all clinical trials. We conducted this systematic review using objective measures such as the Epworth Sleepiness Scale and the Maintenance of Wakefulness Test. Overall, cataplexy was not formally evaluated in the four clinical trials; however, it demonstrated that EDS improved in patients with and without cataplexy. More clinical trials are needed to analyze the efficacy of solriamfetol on cataplexy. The effect of solriamfetol in EDS seems to be conclusive.

## Introduction and background

Narcolepsy is one of the most common disorders that cause chronic sleepiness, affecting around one in 2,000 people [1,2]. The disease has a prevalence in Europe and North America, with an incidence of around 0.03% to 0.05% [2]. The disease affects the sleep-wake cycle in the brain [3]. Excessive sleepiness is caused by a selective loss or dysfunction of orexin (also known as hypocretin) neurons in the lateral hypothalamus [4]. Additionally, the disease may have an immune-mediated component [4].

There are two types of narcolepsy: type 1 and type 2 [2,3]. In type 1 narcolepsy, patients lose around 90% of hypocretin neurons in the hypothalamus, and this results in excessive daytime sleepiness (EDS) and cataplexy [3]. Cataplexy, a unique symptom of narcolepsy type 1, is usually preceded by a strong emotional trigger such as laughter, crying, or stress [3]. Additional clinical features include sleep-wake symptoms such as hallucinations, sleep paralysis, and disturbed sleep [3,4]. In contrast, patients with type 2 narcolepsy do not have significant loss of hypocretin in the brain and have no cataplexy [3].

The Epworth Sleepiness Scale (ESS) is one of the most widely used, validated, self-administered questionnaires for assessing subjective sleepiness over time [2,5]. Patients who present with a score of 11 or greater need further evaluation for a potential sleep disorder [2]. It is essential to confirm the diagnosis of narcolepsy with overnight polysomnography followed by a multiple sleep latency test the next day [1]. Another helpful tool is the Maintenance of Wakefulness Test (MWT), which measures alertness during the day, and can provide complementary, objective measures of the degree of sleepiness [1]. Genetic analyses are not commonly used in clinical practice despite the strong association between HLADQB1 06:02 and narcolepsy type 1, with 97% of patients possessing at least one allele [5].

Historically, some medications have demonstrated efficacy in managing EDS and cataplexy symptoms. However, some patients cannot tolerate them, become refractory, or may use concomitant medications that preclude the use due to drug-drug interaction (DDI) [6]. Therefore, there is a necessity to explore the efficacy of new treatments such as solriamfetol (JZP- 110), a 2019 US Food and Drug Administration (FDA) approved drug [6].

We conducted this systematic review to investigate the efficacy of solriamfetol in EDS through objective measures such as the ESS and the MWT. We aim to investigate the safety and tolerability of solriamfetol and its role in cataplexy.

## Review

Methods

Protocol

We carried out our systematic review following the PRISMA (Preferred Reporting Items for Systematic Reviews and Meta-Analyses) guidelines and* the MOOSE protocol [6].*

Eligibility Criteria and Study Selection

This comprehensive systematic review assumed the following inclusion criteria: all studies written in English and focused on human adults were included. Animal studies and papers that did not meet our research goals were excluded. Eligible designs included clinical trials and observational studies.

Two authors (A.I. and E.P.V.) conducted the screening, each blinded to the other's ratings, and where there was disagreement about study inclusion, a third author, a senior member of the team (J.F.O.), arbitrated. Papers that met the following criteria were included: (1) patients diagnosed with narcolepsy; (2) intervention: solriamfetol; (3) comparator: placebo or control group; and (4) outcomes: ESS, cataplexy rate.

Database and Search Strategy

For this systematic review, studies were selected from PubMed, introducing the search terms: (solriamfetol"[Title/Abstract] AND "narcolepsy"[Title/Abstract]). The search was conducted by J.F.O. between March 1, 2022, and March 15, 2022.

Data Extraction and Analysis

We collected the following information from each study: author and year of publication, the methodology, and the functional outcomes. Baseline characteristics of the study included the number of participants in the treatment, number of participants in the control group, dose, route of administration of the drugs, treatment duration, and timing when the drugs were given. Baseline functional outcomes included the cataplexy rate and ESS.

Bias Assessment

We used the Cochrane collaboration risk-of-bias tool to assess the bias encountered in each study [7].

Results

**Figure 1:**
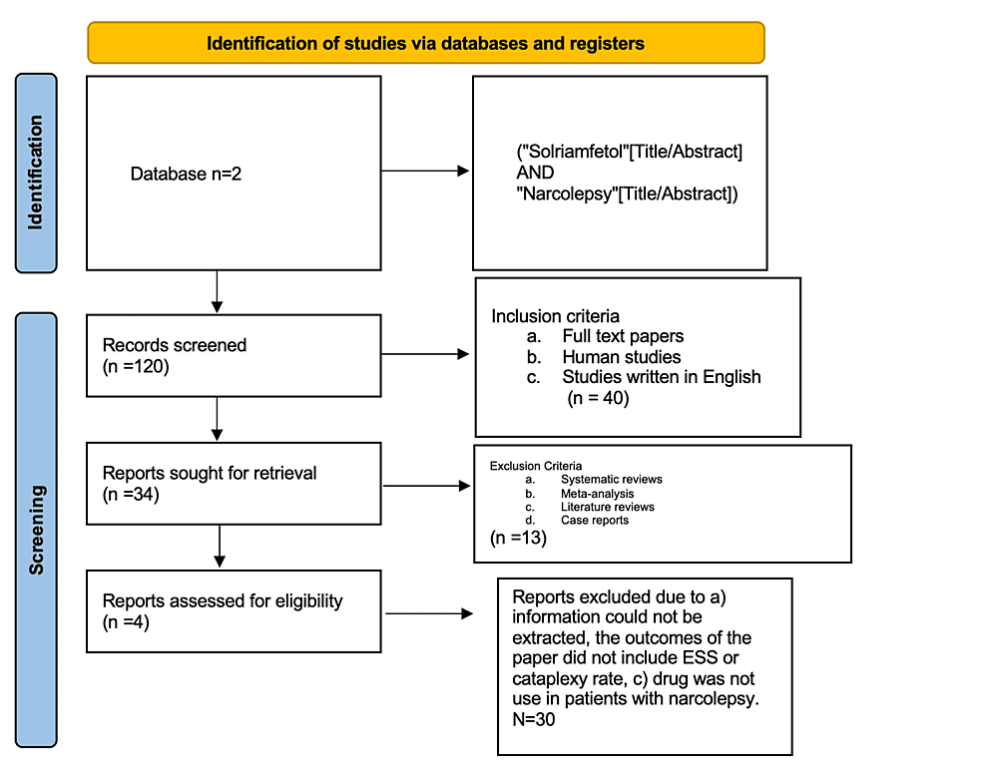
PRISMA flowchart of this systematic review PRISMA, Preferred Reporting Items for Systematic Reviews and Meta-Analyses

Figure 1 shows the PRISMA flow chart of this systematic review.

Study Characteristics

Table 1 shows the study characteristics of the four clinical trials related to solriamfetol and narcolepsy [8-11].

**Table 1:** Study characteristics of the four clinical trials related to solriamfetol and narcolepsy CGI-C, clinical global impression of change; EDS, excessive daytime sleepiness; MWT, maintenance of wakefulness test; OSA, obstructive sleep apnea; mg, milligram; PGI-C, patient global impression scale; RW, randomized withdrawal

Author, year, country	Study type	Number of participants in the treatment group	Number of participants in the control group	Intervention	Outcome/conclusion
Thorpy et al., 2019, United States, Canada, Finland, France, Germany, and Italy [8]	Randomized, double-blind, placebo-controlled trial, parallel‐group study	173	58	Oral placebo or solriamfetol 75, 150, or 300 mg once daily for 12 weeks. Patients randomized to the 150 and 300 mg doses received 75 and 150 mg, respectively, on days 1 through 3 of the first week, with the total dose commencing on day 4.	Once‐daily oral dosing of solriamfetol 150 and 300 mg resulted in major improvements in wakefulness and reductions in excessive sleepiness.
Dauvilliers et al., 2020, USA, Canada, Finland, France, Germany, and Italy [9]	Randomized, placebo-controlled, multicenter, parallel-group clinical trial	173	58	Solriamfetol (75, 150, or 300 mg/day) or placebo. Participants were stratified by cataplexy status.	Solriamfetol effectively treated EDS in participants with narcolepsy with or without cataplexy, as indicated by robust effects on MWT, ESS, and PGI-C. The safety profile was similar regardless of cataplexy status.
Malhotra et al., 2020, USA, Canada, Finland, France, Germany, and the Netherlands [10]	Open-label and double-blind, placebo-controlled	Maintenance phase: narcolepsy, n=231; RW phase: solriamfetol, n=139	Maintenance phase: OSA, n=420; RW phase: placebo, n=142	Participants with narcolepsy or OSA who completed previous solriamfetol studies were eligible. A two-week titration was followed by a maintenance phase of ≤ 50 weeks (stable doses: 75, 150, or 300 mg/day.)	In the maintenance phase, clinically meaningful improvements were noted in ESS, PGI-C, and CGI-C. In the RW phase, the least-squares mean change on ESS was 1.6 in participants continuing solriamfetol versus 5.3 in participants who switched to placebo (p < 0.0001).
Ruoff et al., 2015, USA [11]	Double-blind, crossover study, randomized	33	33	Placebo or solriamfetol at 150 mg/day (weeks 1 and 3) increased to 300 mg/day (weeks 2 and 4)	The efficacy of solriamfetol for impaired wakefulness and excessive sleepiness was observed at 150–300 mg/day and as early as one week after initiating treatment.

Overall, solriamfetol reduced EDS with 150 and 300 mg doses across the studies. Patients with and without cataplexy improved their MWT and ESS scales. The drugs were well tolerated across all four studies.

Limitations and Bias Analysis

From the study conducted by Thorpy et al., conclusions about the effect of solriamfetol on cataplexy are limited as it was not designed to evaluate its effects on cataplexy rigorously. It is still under review if the drug's efficacy observed in this study can be maintained without any safety and tolerability issues in the long term [8].

Limitations of the study conducted by Dauvilliers et al. include the lack of adequate power to evaluate cataplexy effects or detect possible treatment-effect modification in the subgroup analyses. There is also a lack of correction for multiplicity, which limits the interpretation of statistical comparison. It is also unclear whether differences in hypocretin levels had any effect on the outcomes of this study as they were not assessed [9].

There were limitations in the study conducted by Malhotra et al. Firstly, the drug solriamfetol was not compared with other wake-promoting agents. Secondly, the data were collected in the context of rigorously performed clinical trials, and the results reported in the study may differ from those observed in clinical practice. Thirdly, The study focused on subjective outcomes reported by patients and was not designed to assess objective outcomes such as the MWT. It was also noted that neurocognitive performance and motor vehicle accident risk from EDS were not assessed [10].

Some limitations of the study conducted by Ruoff et al. should be noted, including that it was of short duration and included only a limited number of patients. The study design did not directly compare the efficacy and tolerability of the 150 mg/day dose versus the 300 mg/day dose. Additionally, neither objective evaluation using polysomnography of nocturnal sleep nor patient-reported outcomes of function or quality of life were included in the efficacy assessments [11].

**Figure 2:**
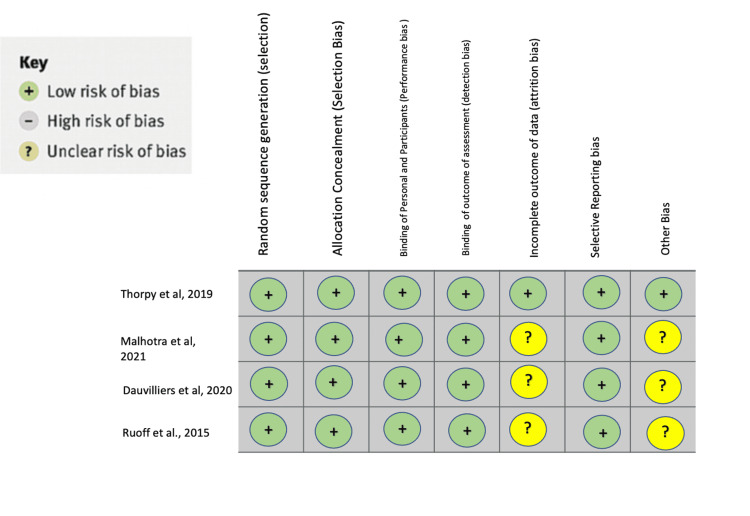
Analysis of bias in this study Thorpy et al. [8]; Dauvillers et al. [9]; Malhotra et al. [10]; Ruoff et al. [11]

Figure 2 shows the analysis of bias in the systematic review [8-11].

Discussion

Solriamfetol is a dopamine and noradrenaline reuptake inhibitor [12]. Solriamfetol does not have monoamine-relaxing properties, which explains why there is a lack of hypersonic when the drug is discontinued [11]. Solriamfetol is classified as a controlled substance class IV due to its abuse potential properties with doses over 300 mg [12]. Overall, in the four analyzed clinical trials, the drug was well tolerated and improved EDS in patients with narcolepsy regardless of whether they have cataplexy.

In the present study, we analyzed EDS and cataplexy. In contrast, other symptoms of narcolepsy, such as hallucinations or sleep attacks, were not analyzed because they were investigated in the clinical trials.

Clinical trials of solriamfetol

Thorpy et al. analyzed the safety and efficacy of solriamfetol in narcolepsy. They found a statistically significant increase in sleep latency compared to placebo using the MWT and ESS scales when solriamfetol dosing was in the range of 150-300 mg. There was no statistical significance for solriamfetol 75 mg compared to placebo [8]. They also found that the higher the dose, the more impact it had on changes from baseline. They found no apparent effect on the number of cataplexy attacks per week, attributed to study limitations. The incidence of adverse effects (AEs) was 68.4% and included headache, nausea, decreased appetite, nasopharyngitis, dry mouth, and anxiety; no serious AEs were reported, and patients with higher doses discontinued the treatment with higher frequency [8]. They concluded that solriamfetol has a safe and tolerable profile and that significant improvements are achieved concerning wakefulness and reduction in excessive sleepiness associated with narcolepsy [8].

Dauvilliers et al. conducted a subgroup analysis of a randomized controlled narcolepsy trial that evaluated the treatment efficacy and safety of solriamfetol in the treatment of narcolepsy in patients with and without cataplexy. They conducted a subgroup analysis by cataplexy status. They found that changes in sleep latency were similar at 12 weeks in patients with or without cataplexy when using MWT and ESS scores [9]. They found that changes in sleep latency were statistically significant in both groups when using doses of 150-300 mg, but they observed more substantial changes in the non-cataplexy subgroup. The ESS scores in the cataplexy subgroup found statistical significance for doses of 150 or 300 mg. In contrast, the differences were statistically significant for doses of 75, 150, and 300 mg [9]. Among the patients who had more than 10 cataplexy attacks per week, the ones who received the doses of 75 and 150 mg had a more significant reduction in attacks compared to the placebo. They concluded that the effectiveness and safety profile was comparable in patients with and without cataplexy [9].

The study by Malhotra et al. evaluated the long-term efficacy and safety of solriamfetol treatment for daytime sleepiness in patients with narcolepsy and obstructive sleep apnea (OSA) [10]. They demonstrated the long-term effectiveness and safety of the drug for up to 50 weeks. They found meaningful clinical improvement in the ESS, patient global impression of change (PGI-C), and clinical global impression of change (CGI-C) scores [10]. They included a controlled randomized withdrawal phase that further provided evidence of efficacy by showing that discontinuation resulted in worsening outcomes. The drug was also well tolerated, but 75% of participants had at least one AE over the study duration. They found no new safety concerns compared to previous 12-week studies [10].

Ruoff et al. evaluated solriamfetol (JZP-110) to treat impaired wakefulness and excessive sleepiness in patients with narcolepsy as assessed by the MWT and ESS. Results showed significant effects at two weeks with solriamfetol 300 mg/day that were statistically significant relative to placebo. Significant treatment effects were also apparent after one week of treatment at a lower dose of solriamfetol (150 mg/day). The oral administration of solriamfetol was well tolerated at both doses, with no serious AEs and no discontinuations due to AEs. The number of AEs reported and the number of patients who reported AEs were similar during the first and second weeks of treatment with 150 and 300 mg/day of solriamfetol, suggesting a lack of dose-dependent AEs within this dose range [11].

New drugs and future directions 

In 2019, pitolisant was also approved by the FDA. Today, pitolisant and solriamfetol are the new drugs used by physicians to treat narcolepsy; other drugs are not appropriately named yet; FT218, JZP-258, and AXS-12 (reboxetine) have completed initial clinical trials and should be in the market soon after FDA approbation [13].

Pitolisant

Pitolisant is an H3 receptor antagonist and inverse agonist that, by blocking histamine autoreceptors, increases the activity of brain histaminergic and noradrenergic neurons, thus helping in the treatment of narcolepsy [14].

According to Fabara et al., pitolisant was studied in four randomized clinical trials where the use of pitolisant, modafinil, and placebo was compared [14]. These showed a significant decrease in ESS in the group that used pitolisant compared to the placebo group. However, its effect was enhanced if combined with other medications [14]. As opposed to solriamfetol, the drug also proved efficacy for cataplexy by reducing the cataplexy rate [14].

Pitolisant was always well tolerated, and in a study by Dauvilliers et al., it was shown that it has a flexible dosage with a high degree of tolerance and minimal AEs [14].

FT218 

Potential treatments for narcolepsy with cataplexy include novel oxybate formulations such as FT218. The modulation of gamma-aminobutyric acid (GABA) B or histamine 3 receptors affects cataplexy and EDS. These drugs, like FT218, have controlled release formulations, and FT218 is currently undergoing phase III trials, where their efficacy is being studied [14]. Studies have also postulated that FT218 would have similar drug-DDIs as sodium oxybate. As with sodium oxybate, the central nervous system (CNS) depressant properties of JZP-258 are shown to have potential interactions with drugs such as benzodiazepines and tricyclic antidepressants (TCAs). Appropriate dose adjustments are recommended in certain situations, such as adding divalproex sodium. While FT218 is effective in treating narcolepsy with or without cataplexy, data from the study also demonstrated weight loss in participants. Being a once-nightly treatment makes it highly advantageous [15].

JZP-258

This formulation combines sodium oxybate, magnesium oxybate, potassium oxybate, and calcium oxybate. The formulation has around 92% less sodium, making it more tolerable. The mechanism is not entirely understood, but it is postulated to be mediated through GABA B modulation during sleep. It has also been approved in cases of idiopathic hypersomnia [15]. During its trial, the most common side effects included GI symptoms, such as nausea and vomiting, and headache. JZP-258 also has DDIs, similar to immediate release formulations of sodium oxybate. As with sodium oxybate, the CNS depressant properties of JZP-258 have potential interactions with drugs such as benzodiazepines and TCAs [15].

AXS-12 (Reboxetine)

AXS-12, also known as reboxetine, has been used in more than 40 countries outside the USA to treat depression since it is a noradrenaline reuptake inhibitor. It affects serotonin reuptake as well. Research on this drug has demonstrated a critical reduction in daytime sleepiness symptoms. With linear pharmacokinetics, it is rapidly absorbed after oral administration. It is highly protein-bound with has a half-life of around 12.5 hours. The drug was mainly metabolized through CYP3A4 [14]. The AEs commonly include dry mouth, constipation, hyperhidrosis, and restlessness. This drug has been recommended as an alternative for patients who cannot tolerate oxybate or pitolisant. Since it is also a CNS depressant, the potential for abuse and misuse is high with classes of medications such as sedatives and hypnotics [15].

## Conclusions

Solriamfetol is one of the newest drugs available to treat narcolepsy. The identified studies show evidence supporting the efficacy, safety, and tolerability of solriamfetol in treating narcolepsy. Solriamfetol demonstrates diminished EDS by reducing the EPS and MWT. It appears to have a dose-dependent effect; the greater the dose, the more the efficacy. The efficacy of solriamfetol seems to be independent of the cataplexy status. This drug does not seem to affect cataplexy, but cataplexy needs to be more formally evaluated in future studies. New drugs have appeared recently, such as pitolisant, an effective drug for EDS and cataplexy. Other drugs such as FT218, JZP-258, and AXS-12 (reboxetine) will be on the market soon.

More clinical trials are necessary to evaluate the efficacy of solriamfetol in cataplexy. Finally, solriamfetol is a controlled substance class IV; thus, it should use carefully due to its risk of abuse potential.
